# Advancements in Varicose Vein Treatment: Anatomy, Pathophysiology, Minimally Invasive Techniques, Sclerotherapy, Patient Satisfaction, and Future Directions

**DOI:** 10.7759/cureus.51990

**Published:** 2024-01-10

**Authors:** Faris Fayyaz, Viralkumar Vaghani, Chukwuyem Ekhator, Muhammad Abdullah, Rashed A Alsubari, Omar A Daher, Danyal Bakht, Hanen Batat, Hareem Arif, Sophia B Bellegarde, Pakeezah Bisharat, Muhayya Faizullah

**Affiliations:** 1 Surgery, Dow University of Health Sciences, Karachi, PAK; 2 McWilliams School of Biomedical Informatics, The University of Texas Health Science Center at Houston, Houston, USA; 3 Neuro-Oncology, New York Institute of Technology, College of Osteopathic Medicine, Old Westbury, USA; 4 General Surgery and Medicine, Jinnah Medical and Dental College, Karachi, PAK; 5 Medicine and Surgery, October 6 University, Raleigh, USA; 6 Obstetrics and Gynaecology, Beirut Arab University, Tripoli, LBN; 7 Medicine and Surgery, Mayo Hospital, Lahore, PAK; 8 Medicine, Yarmouk University, Irbid, JOR; 9 Internal Medicine, Ghulam Muhammad Mahar Medical College, Sukkur, PAK; 10 Pathology and Laboratory Medicine, American University of Antigua, Coolidge, ATG; 11 Internal Medicine, Khyber Medical University, Peshawar, PAK; 12 Medicine and Surgery, King Edward Medical University, Lahore, PAK

**Keywords:** cryotherapy, radioablation, endovenous management, sclerotherapy, varicose seins

## Abstract

Varicose veins are a common vascular condition known for causing discomfort and cosmetic concerns. This comprehensive narrative review delves into their anatomy, pathophysiology, and modern treatment options, with a focus on endovenous techniques and sclerotherapy. The review starts by emphasizing the intricate anatomy of lower extremity venous circulation, underlining the significance of both superficial and deep venous networks in venous return. It also addresses how changes in the venous wall, including valvular insufficiency, contribute to the development of varicose veins. Endovenous techniques like endovenous laser ablation (EVLA), radiofrequency ablation (RFA), and mechanochemical endovenous ablation (MOCA) are explored in detail. These minimally invasive procedures have revolutionized varicose vein treatment, offering high success rates and quicker recovery compared to traditional surgery. The review also highlights their efficacy and safety profiles, aiding clinicians in informed decision-making. Sclerotherapy, a vital modality for varicose veins, is thoroughly examined, covering both liquid and foam sclerotherapy. Foam sclerotherapy, in particular, is recognized for its improved outcomes. The review provides a comprehensive comparison of these treatment modalities, highlighting differences in technical success, recurrence rates, and cost-effectiveness. Patient preferences and satisfaction play a significant role in choosing the right treatment. Safety and potential complications associated with these treatments are explored, with a focus on minor issues and rare adverse events. This review also emphasizes the positive impact of varicose vein interventions on patients' quality of life.

## Introduction and background

Varicose veins are a prevalent manifestation of chronic venous disease, affecting a substantial portion of the global population [1]. These visibly enlarged and tortuous veins, often appearing as protruding purple or blue-green structures on the legs and feet, extend beyond mere cosmetic concerns. They serve as indicators of underlying venous insufficiency, a condition characterized by impaired blood circulation back to the heart. This insufficiency arises due to malfunctioning valves within the affected veins, leading to inefficient blood pumping, retrograde blood flow, and heightened pressure within the veins. While some individuals with varicose veins may remain asymptomatic, others experience localized discomfort, including aching, throbbing, or itching around the affected veins. Over time, more severe symptoms may develop, such as fatigue, heaviness, and leg cramps [2]. If left untreated, chronic venous insufficiency can advance to a more severe stage of venous disease. This progression may entail the emergence of edema (swelling), persistent skin discoloration, eczema (skin inflammation and itching), lipodermatosclerosis (skin and underlying fat hardening), and venous ulcers (open wounds). Estimates indicate that up to 10% of adults with varicose veins may eventually develop advanced venous disease, including venous ulcers, superficial thrombophlebitis (inflammation leading to blood clots), or bleeding from varicosities [3]. It is important to note that the course of chronic venous insufficiency varies among individuals and does not necessarily follow a linear progression [2].

Historically, varicose veins were primarily considered a cosmetic concern, with patient preferences heavily influencing treatment decisions. However, advancements in medical imaging, particularly the adoption of color flow duplex ultrasonography since the 1980s, have revolutionized our comprehension of varicose veins. This technology has enabled a more precise assessment of venous reflux and its association with varicose veins [4]. It is essential to recognize that varicose veins are not merely a benign cosmetic issue but are associated with more substantial health risks [5]. Recent research has shown that individuals with varicose veins face up to a five-fold increased risk of developing deep vein thrombosis (DVT), a potentially life-threatening condition. Moreover, varicose veins have been linked to peripheral arterial disease and other vascular disorders. While certain risk factors for varicose vein formation, such as age, gender, pregnancy, obesity, and prior DVT, are well-established, others remain unconfirmed. Genetic components are also suspected in varicose vein disease, although genetic studies have produced conflicting results [6].

Treatment options for varicose veins range from conservative approaches like compression therapy, lifestyle adjustments, leg elevation, weight management, and medical treatments to interventional methods such as laser thermal ablation, endovenous treatments, and surgery [7]. Surgery, once the standard, has been largely replaced by endovenous thermal ablation (EVTA), offering improved outcomes and fewer complications [8]. Current evidence and guidelines suggest that compression therapy is unnecessary before considering EVTA, although it may be required for insurance purposes [7]. While surgical procedures like vein stripping and ligation are effective, they come with higher complication rates and longer recovery times. Emerging endovenous therapies, including endovenous laser ablation (EVLA), radiofrequency ablation (RFA), steam vein sclerosis (SVS), and endovenous microwave ablation (EMWA), have demonstrated comparable efficacy to surgery in treating varicose veins. Importantly, they offer lower complication rates and shorter recovery times, making them increasingly popular options [8]. Ultrasound-guided foam sclerotherapy (UGFS) serves as a second-line treatment. However, it may not match the long-term success of EVTA methods like EVLA and RFA, particularly in cases with thick-walled veins. Nevertheless, UGFS can be effective in specific scenarios, such as small-diameter or thin-walled veins, making it the optimal choice. Overall, these advancements in treatment options have significantly improved the management of varicose veins [4].

The objective of this review is to thoroughly analyze diverse therapeutic strategies and their efficacy in addressing this prevalent vascular condition. Grasping the intricacies of varicose vein anatomy and pathophysiology becomes imperative. This review will extensively investigate various endovenous techniques, such as EVLA, RFA, and mechanochemical ablation (MOCA), alongside sclerotherapy methodologies, encompassing foam and liquid sclerotherapy. It will meticulously evaluate their mechanisms, relative advantages, and associated complications. Furthermore, this analysis will explore patient satisfaction, quality of life, and the determinants influencing treatment decisions. By engaging in a comprehensive discussion concerning prevailing and emerging treatments, as well as emphasizing safety precautions and outlining future avenues of research, this review aspires to enrich clinical practice, anticipate forthcoming trends in the management of varicose veins, and ultimately elevate the quality of patient care and outcomes.

## Review

Anatomy and pathophysiology of varicose veins

Lower extremity venous blood circulation entails a complex system comprising both superficial and deep venous networks. Within the superficial system, prominent entities include the great saphenous veins (GSV), small saphenous veins (SSV), and their respective tributaries [9]. The GSV courses along the medial aspect of the calf and thigh, eventually merging with the common femoral vein at the saphenofemoral junction (SFJ). Conversely, the SSV traverses the posterior calf, entering the popliteal vein at the sapheno-popliteal junction (SPJ), frequently accompanied by gastrocnemius veins. These systems are intricately interconnected through various tributaries, bearing vital significance in the return of venous blood to the heart [10].

In a normally functioning venous system, the deep venous network significantly contributes to approximately 90% of venous return from the lower limb. The superficial system predominantly receives drainage from the skin and subcutaneous tissues, with a significant portion of this blood seamlessly entering the deep system through perforators situated in the foot, calf, and thigh regions. The venous wall comprises three discernible layers, albeit less distinct compared to arteries. These layers encompass the intima, media, and adventitia, their composition varying depending on vein size and function [10]. As age and pathological conditions progress, all three layers succumb to abnormalities, leading to structural derangement of the venous wall [11].

From a physiological standpoint, venous return against the omnipresent force of gravity hinges upon the muscle pumps in the foot and calf. Contraction of the calf muscles exerts pressure on intramuscular veins, channeling blood into the deep system, subsequently propelling it upwards through the leg. The superficial veins, in turn, serve as collectors of blood from the skin and subcutaneous tissues, facilitating its transference into the deep system during periods of muscle relaxation, mediated by perforating veins. The presence of valves within these veins acts as a barrier, preventing retrograde blood flow during muscle relaxation phases [10]. This valve-mediated closure divides the high-pressure blood column into multiple lower-pressure segments, thereby substantially mitigating venous pooling and capillary hydrostatic pressure, effectively averting edema formation in the lower extremities [12]. However, individuals afflicted with venous insufficiency manifest elevated ambulatory venous pressure (AVP), culminating in the manifestation of symptoms and clinical signs associated with chronic venous insufficiency [10].

Typically, varicose veins are characterized as dilated, tortuous veins exceeding 4 mm in diameter. In contrast, reticular veins represent smaller, nonpalpable dermal veins measuring less than 4 mm in diameter. The histological attributes associated with varicose veins exhibit variability, encompassing irregular intimal thickening, fibrosis, elastic fiber atrophy, collagen fiber thickening, and disarray in muscular layers [13]. These irregularities may display heterogeneity throughout the vein structures [14].

Although reflux serves as the primary hemodynamic aberration in primary venous disorders, it does not singularly instigate the development of varicose veins. Instead, intrinsic structural and biochemical anomalies within the vein wall are postulated to play a pivotal role in their etiology. In secondary venous conditions, the coexistence of both reflux and obstruction is more prevalent than either anomaly in isolation. Limbs exhibiting post-thrombotic skin alterations and ulceration frequently present with a combination of these factors [13]. The specific anatomy of reflux and venous obstruction can significantly influence the severity of chronic venous manifestations, often involving multiple anatomical venous systems, characterizing multisystemic involvement [15]. The emergence of valvular insufficiency subsequent to venous recanalization remains an area of active investigation, with potential mechanisms encompassing thrombus adherence to valve cusps and endothelial erosion [13].

Endovenous techniques

Over the past decade, the landscape of managing symptomatic varicose veins has experienced a substantial transformation, primarily driven by the introduction of minimally invasive endovascular techniques. Among these approaches, EVTA techniques, exemplified by EVLA and RFA, have risen to prominence as the first-line treatments, effectively supplanting conventional surgical interventions for alleviating the discomfort and cosmetic concerns associated with varicose veins [16].

EVLA, pioneered by Dr. Carlos Bone in 1999, has emerged as a pivotal pillar in contemporary varicose vein management. This method entails the insertion of a laser fiber into the targeted vein, emitting laser energy to induce thermal injury within the vessel. The ensuing consequences comprise vein constriction, thrombosis (clot formation), and the development of venous fibrosis. Within the realm of EVLA, investigations into variations in laser wavelengths have been undertaken to enhance effectiveness and mitigate side effects [16]. Radial fibers and lasers characterized by higher wavelengths (such as 1470-1940 nm) have been introduced to promote more uniform damage to the vein wall [17]. Applying a 1470 nm laser in conjunction with a radial probe, for instance, has yielded promising outcomes, marked by reduced post-procedural discomfort and diminished recurrence rates when contrasted with the 940 nm fiber [18]. Nonetheless, the overall success rates for EVLA continue to be notably high, residing at 92% [19].

RFA represents another noteworthy minimally invasive modality for varicose vein management guided by ultrasonography [16]. It harnesses thermal energy delivered via a radiofrequency catheter to ablate the refluxing segment of the vein. During RFA, radiofrequent energy is used to heat the vein wall of the GSV. The catheter is inserted into the vein and direct energy is delivered to the endothelium with the result of collapsing and sealing the vein. One particular device, the ClosureFAST™ RFA system (Medtronic, Dublin, Ireland), has garnered recognition in RFA procedures. The catheter attains temperatures of 120°C during a 20-second treatment cycle, efficaciously sealing the targeted vein [20]. Significantly, RFA has manifested high patient satisfaction and quality of life scores, accompanied by swifter recovery periods relative to traditional surgical interventions [16].

In the comparative analysis between EVLA and RFA, these two modalities exhibit congruous safety profiles and clinical effectiveness. Both offer elevated occlusion rates and expedited resumption of routine activities while exhibiting minimal complications such as thrombophlebitis and hematoma [21]. Long-term follow-up assessments further unveil analogous outcomes regarding venous occlusion rates and patient recuperation. Particularly noteworthy, a decade-long observational study employing a 1470-nm diode laser with radial fibers has substantiated enduring and valuable results for EVLA [17].

MOCA, introduced in 2010 through the ClariVein device (Merit Medical, Utah, United States), introduces a non-thermal and non-tumescent alternative for treating varicose veins. This innovative technique combines mechanical trauma to the vein wall with concurrent injection of a liquid sclerosant, effectively sealing the veins [16]. Polidocanol, also known as Aethoxysclerol®, serves as the sclerosant [20]. MOCA is particularly appealing for addressing veins below the knee and the small saphenous vein, as it mitigates the risk of nerve injury associated with thermal methods such as EVLA and RFA. In a recent multicenter randomized study, MOCA was discerned to be significantly less painful than RFA, rendering it a preferred choice for patients with concerns regarding procedural discomfort. While MOCA may exhibit slightly lower overall success rates compared to other thermal methodologies, it nevertheless represents a valuable alternative characterized by diminished pain and reduced potential for nerve damage [16].

Sclerotherapy

Sclerotherapy represents a versatile medical procedure that assumes a pivotal role in managing varicose veins, addressing a spectrum of venous concerns through the intravenous administration of a chemical sclerosant in either liquid or foam form. This technique is adept at targeting intradermal, subcutaneous, and transfascial veins, as well as epi-, supra-, and subfascial vessels afflicted by venous malformations. The sclerosant's mode of action revolves around the destruction of the vein's endothelium, initiating a transformative process known as sclerosis, ultimately converting the varicose vein into a contiguous string of connective tissue over the long term [22-24].

The primary objective of sclerotherapy does not center on thrombosing the vein, as recanalization may ensue after this phase. Instead, the paramount goal is to transmute the vein into a continuous strand of connective tissue, rendering recanalization an impossibility. This outcome culminates in a functional result commensurate with vein removal or EVTA, thus rendering sclerotherapy a valuable therapeutic option [23].

Certain contraindications necessitate consideration when contemplating sclerotherapy. Absolute contraindications encompass a known hypersensitivity to the sclerosant, acute venous thromboembolism, localized infections in the sclerotherapy region or severe systemic infections, and the presence of a symptomatic right-to-left shunt, particularly pertinent in the context of foam sclerotherapy. Relative contraindications warrant an individualized risk-benefit evaluation and encompass factors such as pregnancy, lactation (with potential discontinuation of lactation for two to three days if urgent treatment is warranted), severe peripheral arterial occlusive disease, compromised general health, a heightened risk of thromboembolism, extended periods of immobility or bedridden patients, and neurological disorders, including migraines, following prior foam sclerotherapy [23].

While liquid sclerotherapy has been in use for a substantial duration, its efficacy, particularly for primary varicose veins, has faced scrutiny due to perceived recurrence rates. However, the advent of foam sclerotherapy has markedly improved outcomes by creating a more extensive interface between the sclerosant and the vein, achieved by incorporating air or carbon dioxide bubbles into the foam [25]. This enhanced methodology engenders improved adhesiveness, heightened echo-visibility attributable to the presence of air, and an amplified sclerosing potential, consequently facilitating reductions in drug dosages and concentrations [26].

The sclerosing agent Aethoxysclerol (with the active ingredient polidocanol) has established itself as a leading choice in sclerotherapy and holds approval for treating both spider veins and varicose veins. Based on currently available data, liquid sclerotherapy is recommended for spider veins and reticular veins. In instances where there are inadequate perforating veins, main or side branch varicosities, recurrent varicosities, pudendal vein varicosis, or venous malformations, foam sclerotherapy has demonstrated its effectiveness [27].

The generation of sclerosant foam entails the application of various techniques, including the Tessari, Monfreux, Frullini, and Cabrera methods [26]. These methodologies yield a mixture of air or carbon dioxide with the liquid sclerosant, with bubble size and sclerosant properties dictating the durability and effectiveness of the foam [28]. Diminutive bubble sizes and elevated sclerosant concentrations within the foam engender superior results. Foam sclerotherapy offers numerous advantages, encompassing efficient displacement of blood, uniform contact of the sclerosant with the endothelium, and provocation of venospasm post injection. When executed meticulously, this approach attains immediate or early closure in medium-to-large veins in over 85% of instances, frequently necessitating multiple sessions for comprehensive success [26].

Sclerotherapy, notably foam sclerotherapy, has garnered favor as a primary modality for refluxing saphenous veins. It is due to its relatively economical nature, feasibility as an outpatient procedure devoid of anesthesia, minimal post-procedural discomfort, and procedural repeatability. Nonetheless, the accurate delineation of indications, selection of the most suitable sclerosant, and utilization of the most efficacious technique remain areas of ongoing exploration. Long-term data relating to quality of life, symptomatic amelioration, and aesthetic outcomes continue to accrue, with ongoing clinical trials striving to furnish more definitive insights into the effectiveness and safety of foam sclerotherapy [26].

Comparison and efficacy

The therapeutic landscape for managing varicose veins encompasses a diverse array of modalities, each endowed with unique attributes and clinical outcomes. An imperative metric, technical success, demonstrates a broadly consistent performance across these modalities. Nonetheless, subtle differentiations in technical success become evident when juxtaposing EVLA with UGFS. EVLA is superior in terms of technical success compared to UGFS. It is paramount to underscore that, although technical success holds substantial significance, an equally critical determinant is the recurrence rate. Existing evidence suggests that, with the exception of a potential long-term benefit for RFA over EVLA, there is generally no substantial variance in recurrence rates among these therapeutic approaches. This suggests that while EVLA may excel in terms of technical success, RFA may potentially demonstrate superior long-term efficacy in preventing the recurrence of varicose veins [29].

The assessment of treatment efficacy transcends mere technical success and encompasses considerations of clinical outcomes and cost-effectiveness. Upon scrutiny of both short-term and long-term clinical outcomes, EVLA emerges as a promising candidate for treating varicose veins. It is proposed as the preferred therapeutic option for eligible patients, as it yields favorable results at the six-month juncture and exhibits promise in cost-effectiveness analyses extending over a projected five-year period. This recommendation aligns harmoniously with the concept that an initial investment in EVLA may yield superior long-term outcomes and engender cost efficiencies [30]. Another pivotal facet to contemplate pertains to the recuperative experience of patients. A comparative study found that the highest rate of technical failures is observed with foam sclerotherapy, while both RFA and foam yielded quicker recovery and less postoperative pain when compared to EVLA and stripping [31].

Cost-effectiveness represents another critical aspect in ascertaining the most appropriate treatment modality. While UGFS initially presents as the most economically efficient option, it often necessitates a higher frequency of subsequent interventions. Day-case surgery, EVLA, and RFA, when performed in outpatient or office-based settings, hold the potential for cost-effectiveness compared to traditional care. These findings provide valuable insights for healthcare providers and policymakers when deciding on the most efficient strategies for managing varicose veins [32]. Furthermore, patient preferences wield substantial influence in the selection of treatment modalities. While a consensus prevails in favor of endothermal ablation for truncal reflux and UGFS for localized and recurrent varicose veins, variances in preferences emerge predicated on factors such as vein dimensions, body mass, leg proportions, and a history of venous thromboembolism (VTE). Patient preferences frequently contemplate the equilibrium between invasiveness, durability, and long-term outcomes. Consequently, the involvement of patients in the decision-making process and the provision of comprehensive information regarding treatment alternatives assume paramount importance [33].

The recurrence of varicose veins after treatment is common, so additional interventional treatment is often necessary. Patients commonly receive multiple interventional modalities within the same treatment session, such as combining EVTA with phlebectomy, or over several sessions, like using laser ablation followed by sclerotherapy for tributary or perforator veins. Relatively high rates of additional interventional treatments have been observed, particularly for EVTA methods like laser ablation or RFA, especially in the short term (52.4% and 40.0%, respectively). Over the course of one year, cumulative rates of additional treatments are notably higher for EVTA techniques compared to reported recurrence rates associated with RFA, laser ablation, and sclerotherapy [34].

Safety and complications

Endovenous techniques and sclerotherapy represent widely employed interventions for managing varicose veins. However, it is imperative to acknowledge that these therapeutic modalities are not devoid of potential complications and adverse effects. EVLA, a valuable option for treating varicose veins, may entail minor complications. Notably, 42.1% of patients undergoing this procedure have reported experiencing erythema or ecchymosis along the path of the long saphenous vein, while 31.6% have complained of induration. Some individuals have also reported paresthesia, limb swelling, and superficial burns. However, significant complications such as DVT and pulmonary embolism (PE) have been infrequent in these cases [35]. In contrast, another study has indicated that EVLA presents a significantly lower incidence of paresthesia compared to RFA and high ligation/stripping, hinting at a potentially more comfortable postoperative experience for patients undergoing EVLA. Nonetheless, thermal skin burns have been observed with comparable frequency in both RFA and EVLA procedures [36].

Although generally efficacious, foam sclerotherapy is associated with a spectrum of potential complications. Notably, anaphylactic/anaphylactoid reactions, albeit exceedingly rare, are recognized as substantial complications necessitating immediate intervention [37]. In addition, though infrequent, extensive tissue necrosis can arise due to inadvertent intra-arterial injection [38]. Skin necrosis represents another uncommon complication, which can emanate from the injection of high-concentration sclerosant or, in rare instances, from the inadvertent intravascular injection of low-concentration sclerosant [39]. Furthermore, foam sclerotherapy has been associated with the potential for transient migraine-like symptoms, with a higher reported frequency compared to liquid sclerotherapy. These symptoms resemble a migraine with aura rather than transient ischemic cerebrovascular events.

More severe complications following sclerotherapy have been documented in rare instances, including stroke and transient ischemic attack (TIA). These events typically manifest after a time interval and are linked to paradoxical thromboembolism. While very rare, instances of DVT and PE have also been reported following sclerotherapy, with the occurrence of DVT being less than 1%. Most DVT cases are asymptomatic and typically detected during follow-up examinations employing duplex ultrasound. Additionally, superficial vein thrombosis, occurring in up to 45.8% of cases, represents another reported complication, although it is predominantly of minor consequence [23].

It is essential to recognize that damage to motor nerves represents an exceedingly low-incidence complication subsequent to sclerotherapy, with an incidence lower than that associated with alternative varicose vein treatment methods [40]. Furthermore, transient general or local reactions may ensue, encompassing symptoms such as chest tightness, vasovagal syncope, nausea, metallic taste, intravascular clot, hematoma, ecchymosis at the injection site, pain at the injection site, local swelling, induration, wheals, blistering, and erythema [23].

Patient satisfaction and quality of life

Varicose veins are a common health issue, primarily affecting adults and often causing a range of uncomfortable symptoms, including pain, swelling, itching, cramps, and a feeling of heaviness. These symptoms not only hinder daily activities but also negatively impact the overall quality of life for those affected. Various factors like age, gender, pregnancy, and lifestyle choices have been recognized as factors that can influence the development and severity of this condition [41]. As a result, the importance of assessing and improving the quality of life (QoL) in individuals with varicose veins has become increasingly emphasized, especially in the context of surgical treatments [42].

Numerous investigations have scrutinized the impact of minimally invasive interventions on the QoL of patients both before and after the procedures, consistently reporting notable enhancements. Patients frequently exhibit a reduction in varicose vein-related symptoms following these interventions, leading to an augmentation of their overall QoL [41]. These findings are congruent with extant literature underscoring the compromised QoL experienced by individuals afflicted with primary and recurrent varicose veins [42]. Moreover, it has been observed that most varicose vein patients encounter difficulties in the execution of their daily activities, often accompanied by deleterious psychological ramifications [43].

Within a specific study under consideration, patients exhibited a significant diminution of symptoms and an augmentation of QoL one month following the minimally invasive procedure. Furthermore, more than half of these patients were able to resume their customary daily routines within the same timeframe, mirroring outcomes from prior research endeavors. Notably, the extant body of literature suggests that minimally invasive interventions not only alleviate symptoms but also contribute to sustained improvements in the QoL of these patients over the long term [41]. Nurses are pivotal in identifying patients at risk by diagnosing symptoms that impact QoL and facilitating early intervention. Their active involvement in the care and management of varicose vein patients is crucial in ameliorating overall well-being [44].

In an investigation focusing on UGFS, the principal finding underscores that UGFS engenders a significant amelioration of lower limb symptoms, cosmetic appearance, lifestyle, and interpersonal relationships in the majority of patients. Many patients harbored expectations that their treatment would alleviate lower limb symptoms, and UGFS not only met but frequently exceeded these expectations. Concerning cosmetic enhancements, a noteworthy cohort of patients anticipated an improved leg appearance, and UGFS effectively realized these cosmetic objectives. These favorable physical and cosmetic outcomes translated into an array of lifestyle benefits, including the capacity to don diverse clothing, enhanced work performance, and the ability to partake in more gratifying social and leisure activities for those who desired such pursuits. Collectively, UGFS emerged as an efficacious modality for enhancing the QoL of individuals grappling with varicose veins, rendering it a valuable therapeutic option [45].

Future directions and research

Ensuring long-term effectiveness is a primary objective in all varicose vein treatments. The likelihood of recurrence remains high, as many individuals have a predisposition to develop additional varicose veins even following comprehensive treatment [46]. Recent progress in varicose vein treatment has enhanced safety, effectiveness, comfort, efficiency, and the ability to achieve long-term success [47]. Dermatologists have been instrumental in advancing and introducing new, noninvasive technologies that are employed in the treatment of both cosmetic telangiectasias and more medically significant, larger varicose veins [48].

In recent years, there has been a significant paradigm shift in treating varicose veins, characterized by the emergence of novel technologies that offer enhanced patient experiences and improved clinical outcomes. Traditional approaches such as vein stripping have given way to a new era of minimally invasive techniques, ushering in remarkable advancements in patient care. One noteworthy innovation is the widespread adoption of EVTA. Typically, this procedure is carried out using either EVLA or RFA. However, alternative methods for EVTA also exist, including steam vein sclerosis (SVS) and EMWA. Non-thermal catheter-based techniques have also gained prominence, notably MOCA and cyanoacrylate glue (CAG). These modalities are often conducted on an outpatient basis under local anesthesia, allowing patients to promptly return home with minimal risk of complications, including DVT. These techniques are particularly advantageous when veins are closely juxtaposed with nerves, mitigating concerns about thermal-related damage [4]. High-intensity focused ultrasound (HIFU) stands out as an innovative technology for managing incompetent N2 truncal veins and incompetent perforating veins (IPVs). HIFU leverages precise, non-invasive ultrasound energy to ablate targeted tissue, exemplified by the SONOVEIN® machine (Theraclion, Malakoff, France). This breakthrough underscores the potential of non-thermal approaches in varicose vein treatment, offering patients a precise and comfortable alternative [49]. Furthermore, some practitioners have explored pioneering hemodynamic approaches such as conservative and hemodynamic treatment of ambulatory venous insufficiency (CHIVA) and ambulatory selective varices ablation under local anesthesia (ASVAL). While these methods may not be universally adopted across all regions, they exhibit promise in delivering effective solutions for varicose veins by addressing venous insufficiency and blood reflux [50,51]. As ongoing research continues to assess their long-term outcomes, these innovative modalities are poised to reshape the landscape of varicose vein management, offering patients a brighter future with enhanced treatment options and improved QoL [47].

## Conclusions

Varicose veins, a prevalent manifestation of chronic venous disease, affect a significant portion of the global population. These enlarged and twisted veins, beyond their cosmetic implications, serve as indicators of underlying venous insufficiency, a condition characterized by compromised blood flow due to faulty vein valves. While some individuals remain asymptomatic, others experience discomfort such as pain, itching, and throbbing. If left untreated, chronic venous insufficiency can progress to more severe stages, leading to complications like edema, skin changes, ulcers, and bleeding. Recent advancements in treatment have enhanced safety, effectiveness, and long-term success.

Dermatologists have played a pivotal role in developing noninvasive technologies for managing both cosmetic and medically significant varicose veins. Treatment options encompass conservative measures and minimally invasive interventions. EVTA techniques like EVLA and RFA have largely replaced surgery due to superior outcomes and fewer complications. UGFS is a valuable second-line treatment, particularly for smaller veins. Ongoing research explores genetics, venous tissue engineering, and stem cell therapy for potential future treatments.

## References

[1] de Ávila Oliveira R, Riera R, Vasconcelos V, Baptista-Silva JC (2021). Injection sclerotherapy for varicose veins. Cochrane Database Syst Rev.

[2] (2021). Nonthermal endovenous procedures for varicose veins: a health technology assessment. Ont Health Technol Assess Ser.

[3] Piazza G (2014). Varicose veins. Circulation.

[4] Whiteley MS (2022). Current best practice in the management of varicose veins. Clin Cosmet Investig Dermatol.

[5] Beebe-Dimmer JL, Pfeifer JR, Engle JS, Schottenfeld D (2005). The epidemiology of chronic venous insufficiency and varicose veins. Ann Epidemiol.

[6] Fukaya E, Flores AM, Lindholm D, Gustafsson S, Zanetti D, Ingelsson E, Leeper NJ (2018). Clinical and genetic determinants of varicose veins. Circulation.

[7] Raetz J, Wilson M, Collins K (2019). Varicose veins: diagnosis and treatment. Am Fam Physician.

[8] Nael R, Rathbun S (2009). Treatment of varicose veins. Curr Treat Options Cardiovasc Med.

[9] Caggiati A, Bergan JJ, Gloviczki P, Eklof B, Allegra C, Partsch H (2005). Nomenclature of the veins of the lower limb: extensions, refinements, and clinical application. J Vasc Surg.

[10] Bradbury AW (2011). Pathophysiology and principles of management of varicose veins. Mechanisms of Vascular Disease: A Reference Book for Vascular Specialists [Internet].

[11] Xiao Y, Huang Z, Yin H, Lin Y, Wang S (2009). In vitro differences between smooth muscle cells derived from varicose veins and normal veins. J Vasc Surg.

[12] Suzuki M, Unno N, Yamamoto N (2009). Impaired lymphatic function recovered after great saphenous vein stripping in patients with varicose vein: venodynamic and lymphodynamic results. J Vasc Surg.

[13] Meissner MH (2005). Lower extremity venous anatomy. Semin Intervent Radiol.

[14] Lowell RC, Gloviczki P, Miller VM (1992). In vitro evaluation of endothelial and smooth muscle function of primary varicose veins. J Vasc Surg.

[15] Hanrahan LM, Araki CT, Rodriguez AA, Kechejian GJ, LaMorte WW, Menzoian JO (1991). Distribution of valvular incompetence in patients with venous stasis ulceration. J Vasc Surg.

[16] Gao RD, Qian SY, Wang HH, Liu YS, Ren SY (2022). Strategies and challenges in treatment of varicose veins and venous insufficiency. World J Clin Cases.

[17] Pavei P, Spreafico G, Bernardi E, Giraldi E, Ferrini M (2021). Favorable long-term results of endovenous laser ablation of great and small saphenous vein incompetence with a 1470-nm laser and radial fiber. J Vasc Surg Venous Lymphat Disord.

[18] Arslan Ü, Çalık E, Tort M (2017). More successful results with less energy in endovenous laser ablation treatment: long-term comparison of bare-tip fiber 980 nm laser and radial-tip fiber 1470 nm laser application. Ann Vasc Surg.

[19] Malskat WS, Engels LK, Hollestein LM, Nijsten T, van den Bos RR (2019). Commonly used endovenous laser ablation (EVLA) parameters do not influence efficacy: results of a systematic review and meta-analysis. Eur J Vasc Endovasc Surg.

[20] van Eekeren RR, Boersma D, Holewijn S, Vahl A, de Vries JP, Zeebregts CJ, Reijnen MM (2014). Mechanochemical endovenous ablation versus radiofrequency ablation in the treatment of primary great saphenous vein incompetence (MARADONA): study protocol for a randomized controlled trial. Trials.

[21] He G, Zheng C, Yu MA, Zhang H (2017). Comparison of ultrasound-guided endovenous laser ablation and radiofrequency for the varicose veins treatment: an updated meta-analysis. Int J Surg.

[22] Rabe E, Breu FX, Cavezzi A (2014). European guidelines for sclerotherapy in chronic venous disorders. Phlebology.

[23] Rabe E, Breu FX, Flessenkämper I (2021). Sclerotherapy in the treatment of varicose veins : S2k guideline of the Deutsche Gesellschaft für Phlebologie (DGP) in cooperation with the following societies: DDG, DGA, DGG, BVP. Hautarzt.

[24] Connor DE, Cooley-Andrade O, Goh WX, Ma DD, Parsi K (2015). Detergent sclerosants are deactivated and consumed by circulating blood cells. Eur J Vasc Endovasc Surg.

[25] Seyam OA, Elshimy AS, Niazi GE, ElGhareeb M (2020). Ultrasound-guided percutaneous injection of foam sclerotherapy in management of lower limb varicose veins (pilot study). Egypt J Radiol Nucl Med.

[26] Subramonia S, Lees TA (2007). The treatment of varicose veins. Ann R Coll Surg Engl.

[27] Lorenz MB, Gkogkolou P, Goerge T (2014). Sclerotherapy of varicose veins in dermatology. J Dtsch Dermatol Ges.

[28] Frullini A, Cavezzi A (2002). Sclerosing foam in the treatment of varicose veins and telangiectases: history and analysis of safety and complications. Dermatol Surg.

[29] Whing J, Nandhra S, Nesbitt C, Stansby G (2021). Interventions for great saphenous vein incompetence. Cochrane Database Syst Rev.

[30] Brittenden J, Cotton SC, Elders A (2015). Clinical effectiveness and cost-effectiveness of foam sclerotherapy, endovenous laser ablation and surgery for varicose veins: results from the comparison of laser, surgery and foam sclerotherapy (CLASS) randomised controlled trial. Health Technol Assess.

[31] Rasmussen LH, Lawaetz M, Bjoern L, Vennits B, Blemings A, Eklof B (2011). Randomized clinical trial comparing endovenous laser ablation, radiofrequency ablation, foam sclerotherapy and surgical stripping for great saphenous varicose veins. Br J Surg.

[32] Gohel MS, Epstein DM, Davies AH (2010). Cost-effectiveness of traditional and endovenous treatments for varicose veins. Br J Surg.

[33] Campbell B, Chinai N, Hollering P, Wright H, McCarthy R (2017). Factors influencing the choice of treatment modality for individual patients with varicose veins. Ann R Coll Surg Engl.

[34] Mallick R, Raju A, Campbell C, Carlton R, Wright D, Boswell K, Eaddy M (2016). Treatment patterns and outcomes in patients with varicose veins. Am Health Drug Benefits.

[35] Chapagain D, Shrestha KP, Thapa Magar D, Shrestha KB, Yadav PK (2021). Recurrence of varicose vein after endovenous laser therapy in a tertiary care center: a descriptive cross-sectional study. JNMA J Nepal Med Assoc.

[36] Dermody M, O'Donnell TF, Balk EM (2013). Complications of endovenous ablation in randomized controlled trials. J Vasc Surg Venous Lymphat Disord.

[37] Cavezzi A, Parsi K (2012). Complications of foam sclerotherapy. Phlebology.

[38] Parsi K, Hannaford P (2016). Intra-arterial injection of sclerosants: Report of three cases treated with systemic steroids. Phlebology.

[39] Goldman MP, Sadick NS, Weiss RA (1995). Cutaneous necrosis, telangiectatic matting, and hyperpigmentation following sclerotherapy. Etiology, prevention, and treatment. Dermatol Surg.

[40] Zipper SG (2000). Peroneal nerve damage after varicose vein sclerotherapy with ethoxysclerol. Single case description with malpractice relevant questions [Article in German]. Versicherungsmedizin.

[41] Tuncer Çoban P, Dirimeşe E (2019). Evaluation of quality of life after minimally invasive varicose vein treatment. Turk Gogus Kalp Damar Cerrahisi Derg.

[42] Beresford T, Smith JJ, Brown L, Greenhalgh RM, Davies AH (2003). A comparison of health-related quality of life of patients with primary and recurrent varicose veins. Phlebology.

[43] Mallick R, Lal BK, Daugherty C (2017). Relationship between patient-reported symptoms, limitations in daily activities, and psychological impact in varicose veins. J Vasc Surg Venous Lymphat Disord.

[44] Kelechi T, Bonham PA (2008). Lower extremity venous disorders: implications for nursing practice. J Cardiovasc Nurs.

[45] Darvall KA, Bate GR, Sam RC, Adam DJ, Silverman SH, Bradbury AW (2009). Patients' expectations before and satisfaction after ultrasound guided foam sclerotherapy for varicose veins. Eur J Vasc Endovasc Surg.

[46] Campbell B (2014). New evidence on treatments for varicose veins. Br J Surg.

[47] Sadick NS (2005). Advances in the treatment of varicose veins: ambulatory phlebectomy, foam sclerotherapy, endovascular laser, and radiofrequency closure. Dermatol Clin.

[48] Sadick NS (2006). Advances in the treatment of varicose veins: ambulatory phlebectomy, foam sclerotherapy, endovascular laser, and radiofrequency closure. Adv Dermatol.

[49] Whiteley MS (2020). High intensity focused ultrasound (HIFU) for the treatment of varicose veins and venous leg ulcers - a new non-invasive procedure and a potentially disruptive technology. Curr Med Res Opin.

[50] Franceschi C (1989). The conservative and hemodynamic treatment of ambulatory venous insufficiency [Article in French]. Phlebologie.

[51] Pittaluga P, Chastanet S (2017). Treatment of varicose veins by ASVAL: results at 10 years. Ann Vasc Surg.

